# The effect of physical exercise on Chinese college students’ mental sub-health: the mediating role of mental resilience and the moderating role of self-efficacy

**DOI:** 10.3389/fpsyg.2025.1572974

**Published:** 2025-06-20

**Authors:** Shuaishuai Zhang

**Affiliations:** ^1^College of Physical Education, Jining University, Qufu, China; ^2^International College, Philippine Christian University, Manila, Philippines

**Keywords:** mental sub-health, physical exercise, mental resilience, self-efficacy, college students

## Abstract

**Objectives:**

This study aimed to explore the influence mechanism of physical exercise (PE) on the mental sub-health (MSH) of Chinese college students, elucidate the mediating pathway of mental resilience (MR) between exercise behavior and psychological state, and test the moderating effect of self-efficacy (SE). This research provides a theoretical basis for optimizing mental health intervention strategies in colleges and universities.

**Methods:**

Utilizing the MSH section of the Adolescent Sub-health Multidimensional Rating Questionnaire (MSQA), along with the Physical Activity Rating Scale (PARS), the Resilience Scale for Chinese Adolescent (RSCA), and the General SE Scale (GSES), a survey was conducted 1,811 students across various academic levels.

**Results:**

(1) PE did not exhibit a direct predictive effect on the MSH of college students; (2) PE affected the MSH of college students through the mediating effect of MR; (3) SE played a moderating role in the relationship between PE and MSH among college students, specifically manifested as follows: With the improvement of the SE levels, the reducing effect of PE on MSH intensified; the reducing effect of MR on MSH also intensified with increasing SE levels.

**Conclusion:**

There was a moderating mediating effect between PE and MSH among Chinese college students. MR served as the mediating factor in this relationship, and SE moderated this effect.

## 1 Introduction

Mental sub-health (MSH) refers to a persistent negative psychological state between mental illness and mental health, characterized by unexplained fatigue, emotional disorders, anxiety, memory decline, and other symptoms. Meanwhile, it was highly concealed and easily overlooked, with a high likelihood of transforming into clinical diseases, such as developing into mental disorders such as depression and anxiety disorders (Shi et al., 2015). In recent years, with the intensification of social competition, increasing academic pressure, lifestyle transformations, and MSH conditions (e.g., anxiety, depression, sleep disorders) had become prevalent among Chinese college students (Su et al., 2021). The “Report on the Mental Health Development of Chinese Citizens (2023–2024)” indicated that the depression level among the 18–24 age group has reached its peak, with the majority of this age group being college students. This highlighted the prominent MSH problem among college students. As college students were in a critical period of physical and mental development, long-term MSH could lead to personality disorders, emotional imbalances, and other issues, seriously affecting their physical and mental health (Liu, 2021). Meanwhile, physical exercise (PE), an important means to promote mental health, had not achieved an ideal popularity rate in China (Ma et al., 2024a). Existing studies have shown that Chinese college students rarely participate in moderate-to-high-intensity physical activities, with widespread insufficient participation in sports and worrying about physical health status (Ma et al., 2024b). This contradictory phenomenon highlights the importance of exploring the relationship between PE and MSH.

Physical exercise (PE) is a planned, organized, and repetitive physical activity aimed at enhancing athletic performance by improving body composition, physical fitness, and athletic ability, with the ultimate or medium-term goal of improving or maintaining physical health (Wang et al., 2024). PE not only increased muscle strength and cardiovascular health (Chekroud et al., 2018) but also had a positive effect on improving the MSH status of college students (Zeng et al., 2021). Existing studies have confirmed that PE can improve an individual’s attention, memory, and cognitive abilities, enhance self-esteem, reduce stress tendencies, help individuals maintain an optimistic mindset, improve mood, effectively reduce depression and anxiety levels, and enhance quality of life (Singh et al., 2023). The higher the degree of PE participation, the lower the MSH score (Philippot et al., 2022). Physical activities have been identified as a promising intervention to improve the mental health outcomes of college students (Ji et al., 2022). At the neurological level, PE promoted the release of happiness hormones such as endorphins, increased brain-derived neural factors, and brought about positive changes in the structure and function (White et al., 2024). From the sociological perspective, PE helped establish social connections, enhance interpersonal communication, and reduce MSH problems (Mahindru et al., 2023). Therefore, it is reasonable to speculate that PE can effectively improve the MSH of college students.

Mental resilience (MR) was a dynamic internal psychological resource that could be acquired through learning and enhanced as individuals continuously overcome adversity (Sisto et al., 2019), serving as an important factor in promoting individual mental health. Richardson’s process model suggested that MR was an important protective factor for mental health: individuals utilize various MR resources to maintain physical and psychological balance, thereby helping college students alleviate psychological discomfort caused by negative events and effectively avoid MSH problems (Richardson et al., 1990). The framework model proposed by Kumpfer posited that an individual’s MR was acquired through the interaction between the individual and the environment (Kumpfer, 2002). PE created an environment where college students could learn strategies to cope with stressful situations and cultivate emotional management abilities during arduous physical training, thereby enhancing their MR, strengthening their willpower, and further improving their mental health level (Toth et al., 2023). Existing studies have regarded PE as an effective intervention for cultivating MR (Qiu et al., 2025). Thus, MR may be an important “bridge” through which PE affects MSH.

Self-efficacy (SE) refers to an individual’s perception or subjective self-assessments of their ability to complete behavioral activities at a specific level (Maddux, 2016). Bandura’s SE theory indicated that the experience of motor mastery was a key influencing factor of SE, and successful experiences of motor mastery had a positive effect on the establishment and consolidation of SE. PE, as an active motor mastery experience, positively influenced an individual’s SE (Bandura, 1982), yet not all college students who participated in PE are free from MSH problems. College students with high SE firmly believe in their ability to complete PE tasks. In the process of achieving this goal, they continuously enhance their self-confidence and sense of achievement, thereby mitigating the occurrence of MSH. Conversely, college students with low SE tended to doubt their ability to complete arduous PE tasks, weakening the predictive effect of PE on MSH (Medrano-Ureña et al., 2020). Empirical research showed that college students with higher MR had better mental health (Wang et al., 2022). However, high MR did not guarantee immunity to MSH, as other factors may intervene (Ding and Liu, 2023). For example, college students with a higher sense of SE generally exhibited stronger MR and fewer MSH symptoms. However, college students with lower SE, despite strong MR, often struggled to overcome feelings of helplessness, tension, and panic when facing pressure, tending to handle problems emotionally, which hindered the application of knowledge and skills, thereby weakening the effect of MR on MSH (Deng et al., 2023). Existing studies have shown that high SE was associated with high levels of subjective well-being, optimism, and life satisfaction, while low SE was linked to more anxiety, pain, and depression symptoms (Ahn and Sim, 2015), indicating that SE had both positive and negative impacts on mental health. Therefore, it is reasonable to infer that SE may moderate the explanatory effect of other factors on MSH. Based on this, introducing SE as a moderating variable is helpful for understanding the interaction mechanism among PE, MR, and MSH.

**Figure 1:**
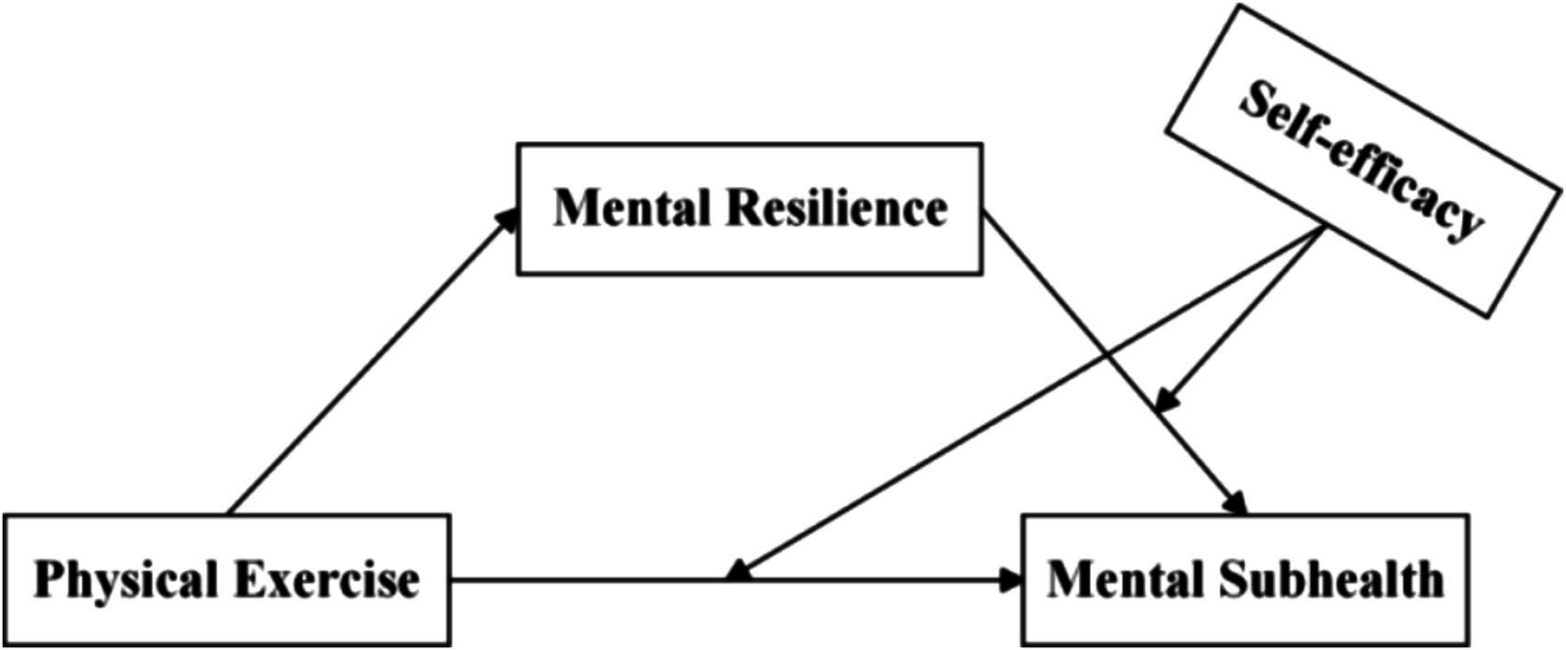
Research hypothesis model.

In conclusion, this study takes the impact of PE on the MSH of college students as the research object and college students as the investigation subjects, aiming to understand the relationships between PE, MR, SE, and the MSH in Chinese college students. Based on previous studies, a moderated mediating model was constructed (Figure 1) to explore “how” (mediating mechanism) and “when” (moderating mechanism) PE affects the MSH of college students, with the aim of further enriching and expanding the influencing factors of the MSH of college students and providing empirical evidence for PE intervention in the MSH of college students. Based on existing research, the following hypotheses were proposed:

*H1*: PE is expected to negatively predict the MSH of college students.

*H2*: MR is expected to play a mediating role between PE and the MSH of college students.

*H3*: SE is expected to play a moderating role in the influence path of PE on MSH.

*H4*: SE is expected to play a moderating role in the influence path of MR on MSH.

## 2 Materials and methods

### 2.1 Participants

In December 2024, a stratified whole cluster random sampling method was employed to select 1,902 college students from seven universities across Zhejiang, Shandong, Jiangsu, Liaoning, Anhui, Fujian, and Guangdong provinces as survey respondents. Stratification was based on academic grade, with assistance from target group counselors to schedule collective testing during class meeting activities. After excluding 91 invalid questionnaires (due to missing responses, excessively short completion times, or high response homogeneity), 1,811 valid questionnaires were recovered, yielding an effective recovery rate of 95.2%. The respondents ranged in age from 18 to 24 years, including 877 males (48.4%) and 934 females (51.6). Class distribution was as follows: 476 freshmen (26.3%), 465 sophomores (25.7%), 437 juniors (24.1%), and 433 seniors (23.9%). This study was approved by the Biomedical Ethics Review of Jining University (2024JNXYLL-035), and all participants provided written informed consent.

### 2.2 Measures

#### 2.2.1 Mental sub-health measurement

The Multidimensional Sub-health Questionnaire of Adolescents (MSQA) (e.g., “*Lack of interest in things*”; “*Often blame oneself*”) was utilized to assess participants’ MSH symptoms. Developed by Tao et al. for adolescent MSH epidemiological research (Tao et al., 2008). It had 39 items with factorial scale components, including emotional problems, conduct problems, and difficulties in social adaptation. A six-point Likert scale was used: 6 = “Duration of more than 3 months”; 5 = “Duration of more than 2 months”; 4 = “Duration of more than 1 month”; 3 = “Duration of more than 2 weeks”; 2 = “Duration of more than 1 week”; 1 = “None or duration of less than 1 week.” Higher total scores indicated more severe sub-health, with no reverse-scored items. In this study, the Cronbach α coefficients of the MSQA scale were 0.96, and the Cronbach α coefficients of each dimension were 0.93, 0.88, and 0.88, respectively.

#### 2.2.2 Physical exercise measurement

The Physical Exercise Rating Scale (PARS-3) (e.g.,*“How intense is your PE?*”*;“How many minutes at a time do you engage in the above-mentioned intense sports activities?”*) was adopted, originally developed by Japanese scholar Hashimoto (1990) and revised by Liang (1994) of Wuhan Institute of Physical Education. This scale evaluated exercise intensity, duration, and frequency using a 5-point Likert scale (1–5). The total score of PE was the product of the scores of the three items, with higher scores reflecting greater exercise participation. The Cronbach α coefficients of the questionnaire was 0.70.

#### 2.2.3 Mental resilience measurement

The Resilience Scale for Chinese Adolescents (RSCA) (e.g., “*I think adversity has an inspiring effect on people.”; “I find it very difficult to control my unpleasant emotions.”*)assessed the MR of the participants. Developed by Hu et al., this questionnaire assessed the MR and adaptability of college students in the face of difficulties and challenges (Hu and Gan, 2008). A five-point Likert scale was used: 1 = “Completely inconsistent”; 2 = “Comparison does not match” 3 = “Unclear” 4 = “relatively consistent”; 5 = “Completely consistent.” It had 27 items with factorial scale components, including goal focus, interpersonal assistance, family support, emotional control, and positive cognition. Among them, items 1, 2, 5, 21, 27, 15, 16, 17, 6, 9, 12, and 26 were scored in reverse, ranging from “completely consistent” to “completely inconsistent” with “1 to 5 points” respectively. Higher total scores indicated better resilience. In this study, the Cronbach α coefficients of the RSCA scale were 0.85, and the Cronbach α coefficients of each dimension were 0.81, 0.73, 0.81, 0.74, and 0.71, respectively.

#### 2.2.4 Self-efficacy measurement

The General Self-efficacy Scale (GSES) (e.g., “*If I try my best to do it, I can always solve the problem*.”; “*I am confident that I can deal with any unexpected things effectively*”) was used to assess participants’ expectations about their ability to perform a certain behavior in a given situation. Developed by German psychologist Schwarzer and Jerusalem (1995) and adapted for Chinese populations by Wang et al. (2001), the questionnaire consisted of 10 items and is scored on a 4-point Likert scale (1 = “Completely incorrect,” 2 = “Somewhat correct,” 3 = “Mostly correct,” 4 = “Completely correct”). There were no reverse-scoring questions in this scale. The average score of all items reflected SE level, with higher scores indicating greater self-efficacy. The Cronbach α coefficients of the questionnaire was 0.87.

### 2.3 Data analyses

Data analysis was conducted using SPSS 26.0, including common method bias tests, descriptive statistics, and correlation analyses. The mediating and moderating roles of MR and SE were examined using the Process 3.3 macro.

## 3 Results

### 3.1 Common method bias test

To evaluate potential biases from self-reported data, the Harman one-factor test was employed. Exploratory factor analysis identified six distinct factors, each with eigenvalues > 1, and the primary factor explained 31.890% of the variance, which was below the 40% threshold for significant common method bias (Hayes, 2013).

### 3.2 Descriptive statistics and correlation analysis among variables

#### 3.2.1 Preliminary and descriptive results

Table 1 presents the results of descriptive statistics for all the research variables. For PE, the results show that male students had higher mean PE scores than females, with juniors scoring the highest and freshmen the lowest For MSH, the results show that females showed higher mean MSH scores than males, with sophomores scoring the highest and freshmen the lowest. For MR, the results show that males had higher mean MR scores than females, with juniors scoring the highest and freshmen the lowest. For SE, the results show that males exhibited higher mean SE scores than females, with juniors scoring the highest and freshmen the lowest.

**Table 1:** Descriptive statistics of demographic variables *M(SD)*.

		PE	MSH	MR	SE	*n*
Sex	Male	40.72 (34.27)	206.42 (44.92)	75.15 (12.14)	2.65 (0.81)	877
	Female	26.51 (27.54)	213.44 (33.68)	74.98 (10.30)	2.57 (0.73)	934
Grade	Freshman	32.06 (30.69)	210.27 (36.82)	73.81 (10.33)	2.47 (0.74)	476
	Sophomore	33.43 (32.02)	212.33 (37.95)	75.29 (11.78)	2.62 (0.77)	465
	Junior	34.18 (32.61)	210.62 (40.59)	75.95 (11.20)	2.67 (0.79)	437
	Senior	34.02 (31.89)	206.74 (43.31)	75.31 (11.50)	2.67 (0.77)	433

PE, Physical Exercise; MSH, Mental Sub-health; MR, Mental Resilience; SE, Self-efficacy.

#### 3.2.2 Correlation analysis among various variables

Table 2 shows strong positive connections among PE, MR, and SE. On the other hand, MSH showed strong negative correlations with these variables.

**Table 2:** Descriptive statistics results and correlation matrix for each variable.

Variable	*M*	*SD*	1	2	3	4
PE	33.39	31.78	–			
MR	75.06	11.23	0.24^**^	–		
SE	2.61	0.78	0.33^**^	0.41^**^	–	
MSH	210.04	39.67	−0.15^**^	−0.47^**^	−0.07^**^	–

PE, Physical Exercise; MSH, Mental Sub-health; MR, Mental Resilience; SE, Self-efficacy.

*p < 0.05, **p < 0.01.

#### 3.2.3 Testing the mediated model with moderation between PE and MSH

**Figure 2:**
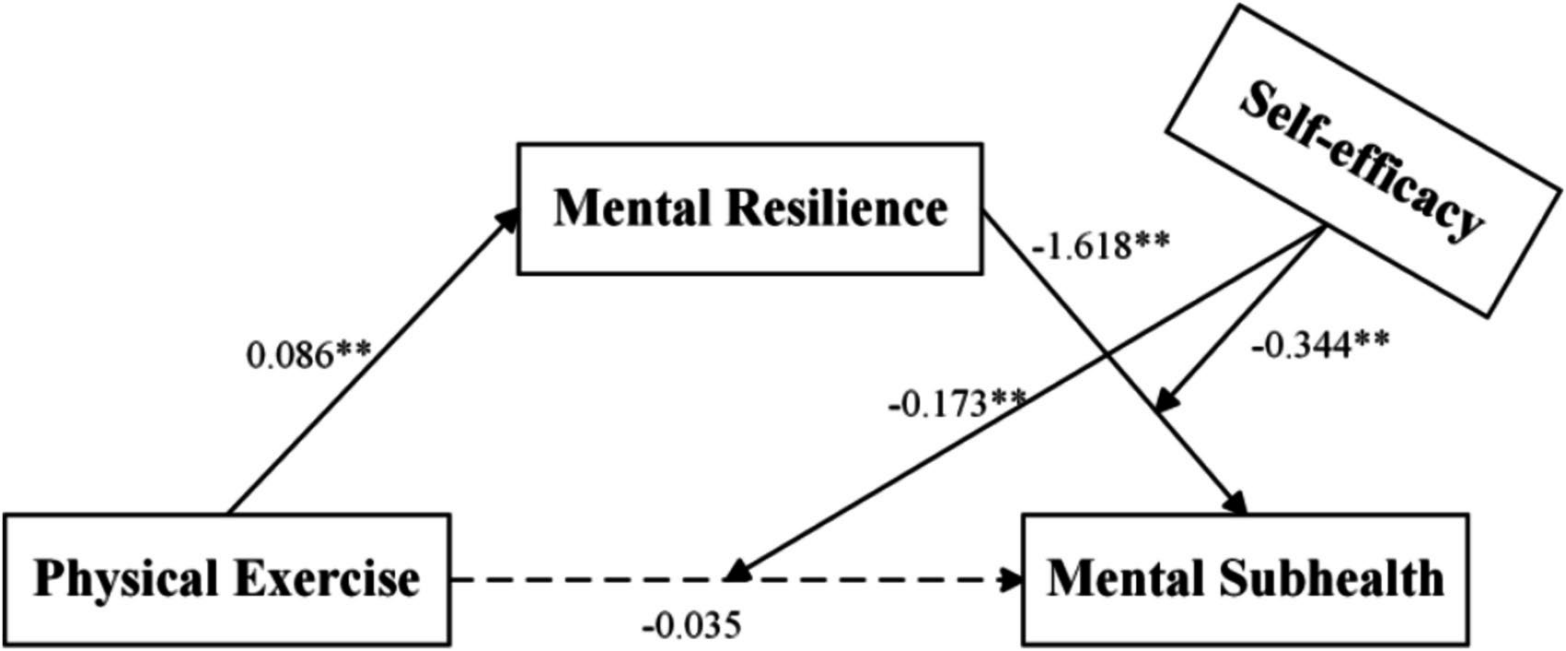
Research result model.

Firstly, a mediating effect test was conducted with PE as the independent variable, MSH as the dependent variable, MR as the mediating variable, besides, sex and grade as the control variables. Structural model evaluations are presented in Figure 2 and Table 3. The mediating effect test of MR was conducted using Model 4 in the SPSS macro program PROCESS developed by Hayes (2017). The results showed that the direct predictive effect of PE on MSH was non-significant (β = −0.035, *t* = −1.260, *p* > 0.05), that is, indicating PE did not directly predict the MSH status of college students. After incorporating MR into the regression equation, PE could significantly and positively predict MR (β = 0.086, *t* = 10.380, *p* < 0.01), and the overall predictive effect of PE on MSH was significant (*F* = 131.114, *p* < 0.01), confirming MR fully mediates the PE-MSH relationship of college students (Table 3).

**Table 3:** Moderating mediating effect test (*n* = 1811).

Regression equation		Overall fit index			Significance of regression coefficient			
Outcome variable	Predictor variable	*R*	*R* ^2^	*F*	β	SE	*t*	95%CI
MSH	Sex	0.474	0.225	131.114	6.409	1.699	3.773	[3.078, 9.741]
	Grade				−0.613	0.740	−0.829	[−2.064, 0.837]
	PE				−0.034	0.027	−1.232	[−0.087, 0.020]
	MR				−1.618	0.075	−21.439	[−1.765, −1.470]
MR	Sex	0.243	0.059	37.800	0.957	0.529	1.809	[−0.081, 1.995]
	Grade				0.428	0.230	1.855	[−0.024, 0.879]
	PE				0.086	0.008	10.380	[0.070, 0.102]
MSH	Sex	0.525	0.276	97.964	6.214	1.644	3.779	[2.988, 9.439]
	Grade				−1.069	0.718	−1.488	[−2.478, 0.340]
	PE				−0.035	0.028	−1.260	[−0.091, 0.020]
	MR				−1.731	0.082	−21.187	[−1.892, −1.571]
	SE				9.167	1.193	7.683	[6.827, 11.507]
	PE × SE				−0.173	0.029	−5.888	[−0.231, −0.116]
	MR × SE				−0.344	0.083	−4.131	[−0.507, −0.181]

Next, a moderated mediating effect test was conducted with PE as the independent variable, MSH as the dependent variable, MR as the mediating variable, SE as the moderating variable, and gender and age as the control variables. Data processing was conducted using Model 15 in the SPSS macro program PROCESS to examine the direct and second-half path of the moderated mediation model (Hayes, 2017). As shown in Table 3, PE still did not directly predict MSH (β = −0.035, *t* = −1.260, *p* > 0.05), but SE strongly predicted MSH (β = 9.02, *t* = 7.56, *p* < 0.01). The PE × SE interaction significantly negatively predicted MSH (β = −0.173, *t* = −5.888, *p* < 0.01), confirming SE moderates the PE-MSH association. For the second-half path, after incorporating the mediating variable MR into the model, MR significantly predicted MSH (β = −1.731, *t* = −21.187, *p* < 0.01), and the MR × SE interaction also negatively predicted MSH (β = −0.344, *t* = −4.131, *p* < 0.01), indicating SE moderates the MR-MSH relationship.

**Figure 3:**
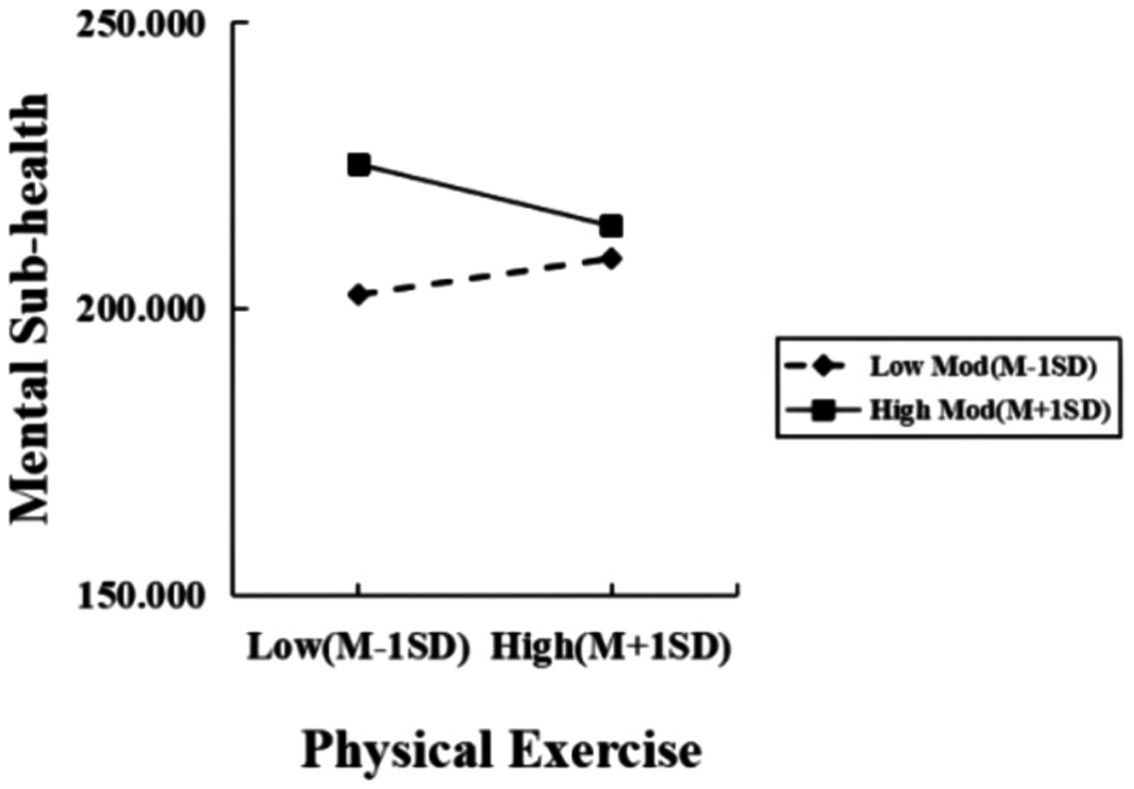
The moderating effect of SE between PE and MSH.

**Figure 4:**
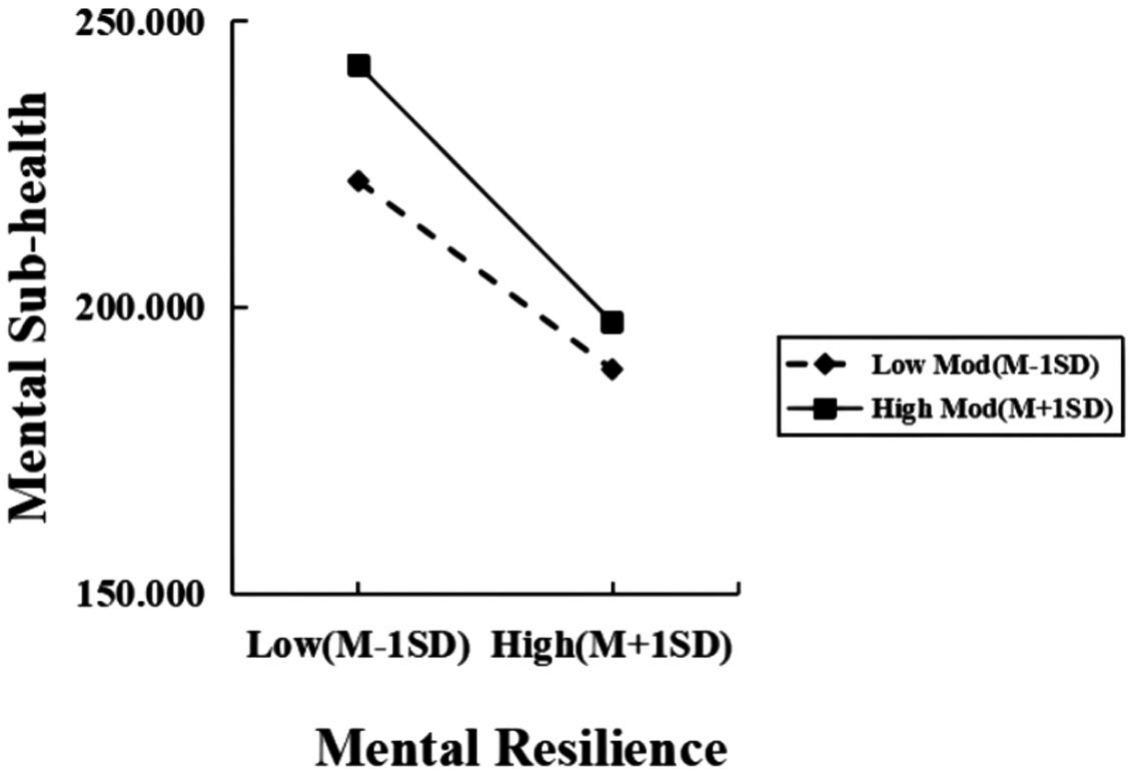
The moderating effect of SE between MR and MSH.

Simple slope tests further analyzed SE’s moderating trend by dichotomizing SE into high/low groups (M ± 1SD) according to one criterion of positive and negative. As shown in Table 4, at low SE (M-1SD), SE significantly moderated PE-MSH (β = 0.099, *t* = 2.484, *p* < 0.05); at high SE (M + 1SD), its regulatory effect on PE and MSH gradually increases, this effect strengthened (β = −0.170, *t* = −5.287, *p* < 0.01), showing high SE amplifies PE’s impact on reducing MSH (Figure 3). Meanwhile, for MR-MSH (M-1SD), low SE groups showed MR’s significant negative prediction (β = −1.465, *t* = −12.462, *p* < 0.01); For college students with high SE (M + 1SD), MR has a greater predictive effect on MSH (β = −1.998, *t* = −22.517, *p* < 0.01), indicating that SE enhances MR’s negative predictive power on MSH (Figure 4).

**Table 4:** Direct effects and mediating effects at different levels of SE.

	SE	Effect	BootSE	95%CI
Direct effect	*M −* 1*SD*	0.099	0.040	[0.021, 0.177]
	*M +* 1*SD*	−0.170	0.032	[−0.233, −0.107]
Indirect effect	*M −* 1*SD*	−0.126	0.020	[−0.168, −0.088]
	*M +* 1*SD*	−0.172	0.028	[−0.229, −0.119]

## 4 Discussion

### 4.1 The role of PE on MSH of college students

This study found that PE negatively correlates with MSH status among Chinese college students. This was consistent with the results of many studies based on the local context of China. It is noteworthy that lots of traditional PE programs are unique to China, such as Tai Chi (Jiang et al., 2020), Five-Animal Exercises (Lun, 2023), and Baduanjin (Zhang and Bayasgalan, 2024), etc. They had an improving effect on negative emotions such as depression and anxiety among college students (Marinelli et al., 2024). Some modern exercise methods, such as sports dance (Zhang et al., 2021), badminton, as well as aerobic training, mixed mode training, etc., all had good effects on improving the MSH status of Chinese college students. This is consistent with the existing research results indicating that regular PE can negatively predict MSH conditions such as depression, anxiety, and stress among college students (Marinelli et al., 2024).

Different PE programs, consistently demonstrate a positive impact on the MSH status of college students. This effect was underpinned by multiple interrelated mechanisms. Neurobiologically, participating in physical activities enhances cognitive function and improves MSH by altering the structural and functional composition of the brain (Pettrey et al., 2024). Exercise can stimulate the growth of new capillaries, which are vital for delivering nutrients to neurons. Neural plasticity, a core component for improving an individual’s mental health, is highly influenced by PE (Roberta et al., 2020). PE promoted the production and release of beta-endorphins in the human body, reduced activities such as adrenaline and cortisol, stimulated cognitive thinking, and enhanced the levels of brain-derived neural factors. These neural factors included the regulation of brain-derived neurotrophic factor (BDNF), vascular endothelial growth factor (VEGF), insulin-like growth factor 1 (IGF-1), etc. (Smith and Merwin, 2021). This is an important biological basis for Chinese college students who have long faced challenges such as high academic pressure and employment competition to enhance their MR and social adaptability.

Psychosocially, participating in group sports activities or sports clubs is an important way for Chinese college students to expand interpersonal communication on campus, build close relationships with peers, and overcome feelings of loneliness. Making like-minded friends and experiencing the joy of sports in sports is particularly important for the group of only-child Chinese college students who lack the support of siblings. Meanwhile, PE can give sports participants a sense of achievement and fulfillment, extending beyond mere physical release. The refreshing joy of exercise fostered social connections with like-minded peers, enhancing interpersonal communication during the process of participating in sports (Johnson and Riley, 2021), thereby reducing the incidence of MSH. PE could enable participants to gain a sense of achievement, not only in terms of physical release and a sense of pleasure, but also in terms of improved body image and self-confidence (Zaccagni and Gualdi-Russo, 2023). Besides, confronting challenges or setbacks in sports strengthens willpower and stress resilience; the process of overcoming obstacles directly enhanced stress-coping abilities (Gubareva et al., 2024). In conclusion, college students’ participation in sports activities has fulfilled their basic psychological needs, such as social connection, autonomy, self-acceptance, environmental control, and life goals. PE can promote interaction with the natural environment, potentially improving mood and alleviating MSH.

In terms of behavioral mechanisms, college students’ participation in sports activities was conducive to sleep at night. Individuals with sufficient sleep showed enhanced emotional regulation and attention stability, which were key to improving MSH (Zhang et al., 2022). Sports participation also fostered coping skill development. Some forms of exercises that combined traditional Chinese programs that united mind and body (e.g., tai chi, martial arts) with modern psychological training methods contained elements of relaxation techniques, positive breathing, and meditation, which in themselves were effective forms of coping skills training (Liu et al., 2024). A systematic review concluded that yoga effectively addressed anxiety or related disorders among Chinese college students (Chen et al., 2025). Because participating in sports activities such as yoga and Chinese martial arts specifically promoted the development of coping skills among college students so as to improve the MSH status of Chinese college students (Sun et al., 2022). In conclusion, in the context of the rapid development of higher education and intensified social competition in China, encouraging and guiding college students to participate in regular PE is an important strategy for cultivating physically and mentally sound and high-quality talents.

### 4.2 The mediating role of MR

This study revealed that MR was the internal mediating mechanism connecting PE and MSH. Specifically, MR exerted a complete mediating role in how PE influenced the MSH of college students: PE did not directly improve the MSH status of college students, but it impacted the MSH of Chinese college students by improving MR. In China, with the popularization of higher education, college students are facing multiple pressures such as academic studies, employment, and interpersonal relationships. Existing studies have confirmed that PE can effectively improve the MR level of Chinese college students (Wu et al., 2020). Notably, higher cumulative PE participation correlated with better MR outcomes (Fan et al., 2024), and PE investment demonstrated a significant positive predictive effect on the MR of college students (Qiu et al., 2025). As a critical protective factor for psychological and behavioral well-being, MR’s healthy development helped individuals mitigate risk factors and maintain mental health (Naden et al., 2023). Recognized as an essential internal capability for navigating challenges, MR was a key indicator of mental health (Gucciardi et al., 2021). Higher MR levels enhanced the ability to buffer negative life events, consequently reducing the likelihood of developing MSH (Lin et al., 2024). In China, PE, as a challenge, can not only improve motor skills but also enhance self-confidence. During the completion process, tenacious perseverance and the courage to constantly overcome challenges are required, which is in line with the spirit advocated in the traditional virtues of the Chinese nation and is conducive to the cultivation and improvement of MR among college students.

Meanwhile, studies had shown that moderate-to-vigorous intensity PE effectively enhanced the MR of college students, enabling them to invest a higher level of effort, perseverance, and behavioral strategies in engaging with PE activities, thereby reducing the incidence of MSH (Belcher et al., 2021). Chinese college students generally face the dual burden of intense academic competition (such as postgraduate entrance examination and employment) and family expectations, leading to MSH issues characterized by “chronic accumulation” rather than “acute onset” of MSH problems. The finding that PE did not directly improve MSH confirms that simple physiological stress release struggles to counteract systemic stress. The complete mediating role of MR indicates that PE can only exert its effect by reshaping an individual’s cognitive framework of stress resistance. For instance, the physical endurance cultivated by college students during long-distance running training can be transformed into psychological endurance to deal with academic setbacks, essentially, elevating the physical experience in sports scenarios into psychological capital, a process highly consistent with the self-cultivation logic of “straining one’s muscles and bones to strengthen one’s will” in traditional Chinese culture. This highlights how college students can cultivate perseverance and enhance their ability to cope with setbacks by participating in PE, thereby improving their MR and promoting the positive development of their mental health.

### 4.3 The moderating role of SE

The research findings reveal self-efficacy (SE) moderates the relationship between PE and MSH. Specifically, this moderation manifests in two ways: Among Chinese college students with low SE, the ameliorative effect of PE on MSH weakens as SE levels decline; conversely, among those with high SE, the positive impact of PE on MSH strengthens as SE improves. In response to this result, the following reasons are analyzed: First, college students with low SE, due to a lack of confidence in their own abilities, hesitate to try new forms of exercise or challenge their own limits. When facing PE, they do not have sufficient motivation and interest, thereby limiting the improvement of exercise effects (Schönfeld et al., 2019). They thought they were not competent for a certain sport or could not achieve the expected sports effect, and thus were reluctant to invest time and energy in participating in PE (Ashford et al., 2010). Physical education courses in Chinese colleges and universities often focus on the mastery of skills and the assessment of grades, which may make some students with low self-efficacy feel stressed and frustrated in PE, further reducing their enthusiasm for participating in PE.

Meanwhile, college students with high SE demonstrate stronger capabilities to cope with various challenges in life, including academic pressure, interpersonal relationships, etc. For example, in sports clubs and teams of Chinese universities, students with high SE are more willing to take on leadership roles and organize and participate in various sports activities. This not only improves their sports skills but also enhances their psychological quality. Furthermore, college students with high SE tend to adopt positive coping strategies when faced with stress and negative emotions. Believing that exercise can help them enhance their emotional state not only provides positive psychological reinforcement, but also boosts exercise’s efficacy in emotional regulation. Furthermore, students with high SE are usually more proactive in seeking social support. For instance, on Chinese university campuses, basketball clubs, running clubs, etc. often have a large number of members. They regularly organize training and competitions, creating a positive and upward sports atmosphere. Students with high self-efficacy are more likely to persist in exercising and gain psychological support and satisfaction in such an atmosphere. These social networks provided them with emotional encouragement and companionship, further enhancing the positive effects of exercise and reducing the risk of MSH (Wang et al., 2022). This aligned with prior findings that “people with a strong sense of SE were considered to make more efforts in pursuing their goals and be more persistent when facing setbacks ordifficulties” (Ghasemi et al., 2017).

In the context of Chinese higher education, where collectivist values and competitive pressure coexist, college students with low-SE are more likely to fall into learned helplessness during PE. Conversely, high-SE students associate exercise goals with academic outcomes when confronting “involution” pressure, exemplified by beliefs such as “exercise boosts learning efficiency.” This forms a unique psychological defense mechanism to achieve stress buffering (Di Maio et al., 2021). Therefore, for college students with high SE, PE plays a more significant role in reducing MSH.

This study also found that SE can regulate the relationship between MR and MSH. Specifically, among both low- and high-SE college students, improving MR correlates with continuous MSH enhancement, while SE amplifies MR’s positive effect on MSH. This finding was explained by the cognitive-behavioral synergy framework. To begin with, at the cognitive level, college students with high SE are more inclined to interpret stressful events as manageable challenges rather than threats. This positive cognitive style worked together with MR to form a “cognitive buffer zone” against MSH (Qin et al., 2023). For instance, when facing academic competition, Chinese college students with high SE are more likely to mobilize resilience resources for goal reconstruction and transform the pressure of “involution” into the motivation for self-improvement. This cognitive process enables MR to exert a “leverage effect” on the improvement of MSH. Conversely, although Chinese college students with low SE still benefited from the improvement of absolute MR levels, more of their cognitive resources were consumed in self-doubt and threat assessment, resulting in a ceiling effect in the utilization efficiency of MR resources (Qin et al., 2023).

Further analysis shows that at the behavioral level, within China’s cultural framework of achievement prioritization, students with high SE are better at transforming MR into specific action strategies, such as actively seeking social support, adhering to a healthy lifestyle, and formulating phased solutions. This transformation efficacy is particularly crucial in the Chinese educational landscape. In collectivist cultural settings, individuals often face behavioral conflicts between “saving face” and “seeking help,” but SE could precisely resolve this contradiction and prompt students to utilize MR resources more flexibly (Mao et al., 2023). College students with high SE transform the quality of MR into specific health-promoting behaviors. This behavioral conversion rate directly amplifies the intervention effect of MR on MSH.

Notably, collectivist characteristics in Chinese culture may further amplify this moderating effect. Under the dual influence of family expectations and social comparisons, Chinese college students with high SE often forms a positive cycle with a sense of responsibility and achievement motivation. This cultural adaptability made the positive effect of MR more likely to be transformed into psychological capital by SE (Meng et al., 2015). Meanwhile, SE effectively blocks the continuous emergence of MSH conditions due to the accumulation of frustration that may be caused by insufficient MR by enhancing an individual’s sense of control over stressful situations. Thus, strengthening SE to enhance MR may serve as a means to improve the mental health of Chinese college students (Mao et al., 2023).

## 5 Implications

The current research offered novel insights into preventing and mitigating MSH among Chinese college students. It confirmed that MR and SE serve as negative predictors of MSH further clarifies the interrelationships among these factors. Meanwhile, considering the Chinese context, the mechanism through which PE influences MSH, suggesting that Chinese universities and physical education instructors can guide students to select appropriate PE programs based on their SE levels and enhance their MR by improving SE. This approach equips students to better handle academic and life pressures, potentially reducing the incidence of MSH.

To address the issue of MSH among Chinese college students, the following suggestions are proposed: (1) Include PE in compulsory courses in Chinese colleges. Design diverse sports exercises regularly to meet the interests and physical needs of different students. Organize sports events, fitness club exercises, and other forms of activities to encourage students to actively participate, not only to improve physical fitness but also as an effective way to improve MR. (2) Promote the integration of mental health education with PE. Chinese colleges should provide mental health education courses, especially on the cultivation of MR and SE, combined with the practice of PE so that students can understand the positive impact of exercise on mental health. Through case studies, group discussions and other forms will help students recognize the role of PE in coping with stress and improving emotional management skills. (3) Provide SE training. Chinese colleges should regularly arrange SE workshops or training courses to teach students how to set achievable goals, overcome difficulties, and be self-motivated. These courses can improve students’ self-confidence and ability to cope with challenges through interactive means such as role-playing and case studies. (4) Build a home-college collaborative education mechanism. Encourage Chinese parents to establish close ties with colleges and participate in students’ PE programs and mental health education. Parents should establish a correct concept of health, support and encourage their children to participate in PE, and the colleges should also provide feedback to parents on a regular basis about students’ PE and mental health status at school, so as to create a good atmosphere of home-college co-education and jointly promote the comprehensive development of students.

## 6 Limitations and future directions

There are some limitations in this study. Firstly, its cross-sectional design does not fully establish the causal mechanisms between PE and MSH in Chinese college students, necessitating future longitudinal studies to more definitively explore these causal relationships. And then, the analysis of PE was limited to categorizations of high and low amounts, so future research should consider varying intensities—low, medium, and high—and examine specific PE regimens to deepen understanding. Therefore, future studies may include longitudinal analysis of individual cases, while also considering the intensity, frequency, and duration of PE in further consideration.

## 7 Conclusion

The current study examined the relationships between PE, MSH, MR, and SC among Chinese college students. The findings revealed that: (1) PE does not directly predict MSH; (2) MR acts as a full mediator in this relationship; (3) SE exerts a negative moderating effect, with high SE amplifying impact of PE on MSH among Chinese college students; (4) SE also negatively moderates the effect of MR on MSH. This moderation is more pronounced in students with high SE.

## Data Availability

The raw data supporting the conclusions of this article will be made available by the authors, without undue reservation.
